# Challenges in assessing the potential allergenicity of biotechnology products

**DOI:** 10.1186/2045-7022-1-S1-S11

**Published:** 2011-08-12

**Authors:** Richard Goodman

**Affiliations:** 1 University of Nebraska-Lincoln, Food Allergy Research and Resource Program, Dept. of Food Science & Technology, Lincoln, NE, USA

## 

Regulatory guidelines for assessing the safety of new food crops developed using biotechnology were published in the early 1990s, before the introduction of genetically modified (GM) crops. Many GM crops are now widely adopted as 77% of global soybean and 26% of maize production is from GM varieties. The early safety assessment guidelines seem to work as there are no proven cases of harm from these products. The greatest potential risk of food allergy would be the transfer of a gene encoding an allergen or protein sufficiently identical to an allergen to a different food crop so that unexpected reactions might occur upon ingestion. These risks can be evaluated using sera from subjects allergic to the source or with a sequence matched to an allergen based on FASTA or BLASTP. Some potential risk is also possible due to de novo sensitization as nearly every food may cause occasional allergies, yet only a few foods and proteins present great risk. The current paradigm considers stability of the protein in pepsin under fixed conditions, abundance of the protein in foods and the potential impact of heating and processing, features common to some major allergens. Those methods are not fully predictive and will likely be improved through experience. However, proposed revisions by the European Food Safety Authority (EFSA) as well as demands from some regulators to include the use of short-amino acid sequence matches, animal models and evaluation of potential changes in endogenous allergen expression using proteomics approaches could reduce the effectiveness of the assessment. The value of new tests should be rigorously tested. It is clear that absolute safety is not possible and that demands for these tests have never been applied to new crop varieties derived through inter-specific hybridization, mutagenesis or the introduction of a whole new food crop.

